# The relationship between body composition and physical fitness in 14 year old adolescents residing within the Tlokwe local municipality, South Africa: The PAHL study

**DOI:** 10.1186/1471-2458-12-374

**Published:** 2012-05-24

**Authors:** Makama Andries Monyeki, Rik Neetens, Sarah J Moss, Jos Twisk

**Affiliations:** 1 Physical Activity, Sport and Recreation Research Focus Area, Faculty of Health Sciences, North-West University, Meyer Street, Potchefstroom 2520, Republic of South Africa; 2 EMGO Institute and Department of Clinical Epidemiology and Biostatistics, VU Medical Medical Center, Van der Boechorstraat 7, Vrije Universiteit, Amsterdam, 1081 BT, Amsterdam, The Netherlands; 3 School of Biokinetics, Recreation and Sport Science Private Bag x6001, Potchefstroom 2520, Republic of South Africa

**Keywords:** Physical fitness, Body composition, Underweight, Overweight, Adolescents, PAHL study

## Abstract

**Background:**

Little is known about the relationship between body composition and physical fitness in 14 year-old high school adolescents of South Africa. Baseline data from a longitudinal study on physical activity and health (PAHLS) may provide valuable information for future studies, hence to inform public health policy makers. The objectives of this study are to determine the prevalence of underweight, normal weight and overweight among adolescents aged 14 years in the Tlokwe Local Municipality of the North West Province of South Africa, and to assess the association between physical fitness and body composition separately for boys and girls, adjusted for race and locality.

**Methods:**

Body weight, height and triceps, and subscapular skinfolds of 256 adolescents (100 boys and 156 girls) aged 14 years were measured, and percentage body fat and body mass index (BMI) were calculated. BMI was used to determine underweight, normal weight and overweight based on the standard criterion. Physical fitness was assessed by standing broad jump, bent arm hang and sit-ups according to the EUROFIT fitness standard procedures. Multinomial logistic regression analyses stratified for gender and adjusted for race (black or white), and the locality (urban or township) of the schools were used to analyze the data.

**Results:**

In the total group 35.9% were underweight and 13.7% overweight. Boys were more underweight (44%) than girls (30.7%). The prevalence of overweight was 8% in boys and 17.3% in girls. BMI was strongly (p = 0.01) related with percentage body fat. Strong and significant positive associations between physical fitness and BMI for the underweight girls with high physical fitness scores (OR, 10.69 [95%CI: 2.81-40.73], and overweight girls with high physical fitness scores (OR, 0.11 [95%CI: 0.03-0.50]) were found. Non-significant weaker positive relationship between physical fitness and BMI for the underweight boys with high physical fitness scores (OR, 1.80 [95%CI: 0.63-5.09]), and the overweight boys with high physical fitness scores (OR, 0.18 [95%CI: 0.02-1.78]) were found.

**Conclusion:**

Both underweight and overweight among boys and girls in Tlokwe Local Municipality exist, and their effects on physical fitness performances were also noticed. As such, strategic physical activity, interventions or follow-up studies recognizing this relationship particularly in the overweight adolescents are needed. In addition, authorities in health and education departments dealing with adolescents should make use of this evidence base information in policies development.

## Background

Epidemiological studies indicate the coexistence of both underweight and obesity as major problems in both developed and developing countries respectively [1-6]. In Sub-Saharan Africa the presence of overweight and obese children and adolescents are reported to vary between 5–17% and 1–5% respectively [3-5]. In South Africa, results from the National Demographics and Health Survey published in 2002, revealed that over 57% of adult South African women were overweight or obese, doubled that of adult South African men, whereas 29% of the population may be classified as either overweight or obese [7,8]. A study in the Potchefstroom area of the North West Province on 12–18 year adolescents reported 8.6% obesity [9]. In the same population the prevalence of underweight was also found to be high in children [10].

Understanding the trends of overweight or obesity and underweight in adolescents is important, because it is associated with adverse effects on health and social repercussion in both adolescence and adulthood [11-14]. It is suggested that adolescence is a crucial period of life, since dramatic physiological and psychological changes take place at these ages as it may constitute the last possible growth spurt [15-17]. During this stage of life the development of physiological health risk factors depends largely on the initiation of health-compromising behavior such as poor eating and inactivity [8,18,19]. Studies during adolescence would add support to the primary assumptions given for early interventions to prevent risk factors of non-communicable diseases before behavioral patterns are fully established and resistant to change [20-24]. The consequences of the adverse health effects of underweight and obesity are likely to be the development of hypokinetic diseases such as hypertension, cancer and Type II diabetes [25-29] as well as reduced health-related physical fitness [30-34]. Excessive fatness (determined by body mass index (BMI) - a useful surrogate of percentage body fat) is found to be negatively associated with performance tasks in which the body is projected through space, as in standing broad jump, and on tasks in which the body must be lifted in space, as in bent arm hang [30-32]. Consequently, hypokinetic diseases as well as poor physical fitness have the potential to place considerable future burden on spiraling health costs and services [27,35]. As such, early identification of adolescents at risk is essential for prevention of adulthood obesity [36].

In South Africa, despite reported high prevalence of underweight and obesity [7,9], scanty information exists regarding the relationship of body composition with physical fitness in 14 year-old adolescents attending high schools in town and township areas within the Tlokwe Local Municipality of South Africa. A study conducted by Kruger et al. [32] reported high prevalence of overweight in urban school girls on the one hand, while on the other side studies by Van Rooyen et al. [10] revealed a high percentage of underweight in township children. Studies which investigate the relationship between body composition and physical fitness in South Africa exist, of which studies that could be found where mostly performed on undernourished children [37,38]. The objectives of this study designed as a baseline initiative within a longitudinal study are therefore in twofold: to determine the prevalence of underweight, normal weight and overweight among adolescents aged 14 years old in the Tlokwe Municipality of the North West Province of South Africa, and to assess the association between physical fitness and body composition separately for boys and girls adjusted for race and locality.

## Methods

### Study area

This study was conducted in Tlokwe Local Municipality (previously known as Potchefstroom Municipality) of the Dr Kenneth Kaunda District Municipality in the North West Province, South Africa. The Tlokwe Local Municipality is located between 26° 43′ 0″ South and 27° 6′ 0″ East, and longitudes 27,1000 (276′0.000″E). The municipality encompasses several neighboring settlements with a population of 128,357 in a density of 48 km^2^ (124.2 sqm), according to the 2007 community survey. The area is primarily inhabited by Black Africans (_˜_70%), 27.0% White Africans, 3.0% Colored and 0.4% Asian [39]. The major language spoken in the area is Setswana, Afrikaans and English. The seat of the local municipality is Potchefstroom.

The major staple food is maize meal, which is eaten by the majority of South Africans. It is very popular as breakfast porridge or as “pap” with a “braai” (barbeque). Fruits and vegetables are also grown or are available in store markets, and eaten in small quantities. Beef and chicken are the main source of protein. Fast food outlet stores are accessible in almost all corners of the Potchefstroom town area [39].

### Sample

Data for this study were obtained from a baseline survey which is part of an observational multidisciplinary longitudinal study on physical activity and health (PAHLS) that started in 2010 and will be continued for a period of five years. The purpose of this study is to determine the relationship between body composition and physical fitness in 14 year adolescents residing within the Tlokwe Local Municipality of the North West Province of South Africa. It is believed that the largest percentage of variation in performance accounted for by chronological age, skeletal age and body size generally occur at age 14, in which height and weight are found to be interrelated [19], hence the present study was conducted on 14 year adolescents.

The study involved six schools from a total of eight schools within Tlokwe Local Municipality which were initially randomly recruited to participate in the study. The participants were requested to provide demographic information in terms of their gender status, race and locality (i.e. town or township). For the purpose of this study, school-based locality comprised of four schools from town and four schools located in township areas. The included schools covered both low and high socio-economic circumstances of pupils. The South African Department of Education categorize schools in quintiles (1–5) according to physical condition, facilities and crowding and the relative poverty of the community around the schools [40]. Out of the eight schools initially selected, two urban schools refused to participate (without providing reasons). In line with the general aim of PAHLS which investigate over a five (5) year period a longitudinal development of physical activity and determinants of health risk factors of health behavior in 14 year-old adolescents living in the North West Province of South Africa, in all selected schools children who were 14 years old were eligible to participate in the study and were recruited. School records as well as participants’ birth clinic cards were used to establish the age of the participants in the study. Only healthy children whose parents gave consent were allowed to participate in the study. A total of 256 adolescents (100 boys and 156 girls) from six schools completed the anthropometric measurements and physical fitness tests. For the purpose of this study, locality of the schools (i.e. town and township) was used for designation. The measurements for this study were collected from April to May 2010. The participating schools were briefed about the purpose of the study, and subsequently written informed consent was obtained from the school authorities, the parents and the pupils of the participating schools. Permission for the study was granted by the District Manager of the Department of Education in Potchefstroom. In addition, the study also received approval from the Ethics Committee (*Ethics no: NWU-0058-01-A1*) of the Potchefstroom Campus of the North-West University.

### Anthropometric measurements

Anthropometric measurements of height, weight and skinfolds were measured by Level 2 Criteria according to the standard procedures described by the International Society for the Advancement of Kinanthropometry: ISAK [41]. Height was measured by the use of stadiometer to the nearest 0.1 centimeters (cm) with participants in a bare feet standing upright position with the head in the Frankfort plane. Weight was measured to the nearest 0.1 kilogram (kg) with an electronic scale with the subject wearing minimal clothing. The triceps and subscapular skinfolds were measured to the nearest 0.2 mm with a Harpenden (British Indicators, UK) skinfold caliper, and the average of two measurements was used. Body mass index (BMI) as a measure of body composition was calculated as body mass/stature² (kg/m²). Subsequently, normal weight and overweight/obesity as well as underweight/thinness were classified according to the age- and sex-specific cut-off points for children according to Cole et al. [42] and Cole et al. [43] respectively. Percentage body fat (%BF) also as a measure of body composition was based on the sum of triceps and subscapular skinfolds derived from skinfolds using the equation developed by Slaughter et al. [44], which is internationally accepted for use in children and adolescents from different ethnic groups.

### Physical fitness tests

The physical fitness included standing broad jump, sit-ups and bent arm hang from the European Test of Physical Fitness [45]. Standing broad jump is designed to measure explosive strength and the results are expressed in centimeter. Bent arm hang is designed to measure static arm strength and is expressed in seconds. Sit-ups, designed to measure functional strength, is expressed in the number of situps in 30 seconds.

### Statistical analysis

Descriptive characteristics (mean, standard deviations and frequencies) were calculated for body composition and physical fitness measures separately for the total group, and for boys and girls. A one-way analysis of variance (ANOVA) was used to test differences in the body composition and physical fitness characteristics among the weight categories. Scheffe post hoc analysis was used to locate the differences among the three BMI category groups. Multinomial logistic regression analysis (odds ratios; (OR)) was used to study the relationship between level of physical fitness and BMI categories. Level of physical fitness was analyzed as a determinant for the BMI categories. Each of these variables were first grouped into four groups, where the groups were scored from low to high scores on the physical fitness variable (scored as 1–4 respectively). Generating the groups was done by using Rank Cases (Ntiles) in SPSS (SPSS Inc). By adding up the group scores the combined scores of physical fitness were calculated. These combined scores were also used for grouping using Rank Cases. From the combined scores three groups were composed; low physical fitness, moderate physical fitness and high physical fitness. In the analysis low physical fitness was used as the reference group. For the BMI categories, normal weight was used as the reference group. The analysis was stratified for gender and locality (i.e. urban or township) of the schools and race (i.e. black or white) were checked for confounding. Statistical significance was set at P < 0.05 and all analyses were performed using SPSS version 17.

## Results

The characteristics of the study sample are presented in Table1. They were 100 boys and 156 girls entering high schools from town and township areas within the Tlokwe Local Municipality. The mean age was about 14.02 years for boys and 13.92 years among girls with no significant age differences (p = 0.13), however most traits differed by gender. BMI was strongly (p = 0.01) related to %BF. The prevalence of underweight, normal and overweight/obese are also summarized in Table1. The prevalence of underweight is 35.9% and overweight 13.7% for the total group sample. When analyses were performed separately by gender, boys showed higher prevalence of underweight (44%) than the girls (30.7%) on the one side, while on the other hand girls showed higher prevalence of overweight/obesity (17.3%) than boys (8%). No significant differences were found for locality and race.

**Table 1:** Characteristics of the sample by total group and gender

	**Total**	**Boys**	**Girls**	**p-value**
	**(n = 256)**	**(n = 100)**	**(n = 156)**	
Age (years)	13.96 ± 0.50	14.02 ±0.59	13.92 ± 0.43	0.134
Body mass (kg)	51.72 ± 12.96	51.00 ± 13.01	52.19 ± 12.91	0.41
BMI (kg/m^2^)	20.71 ± 4.22	19.71 ± 3.62	21.35 ± 4.46	0.020
%BF	17.83 ± 7.01	13.68 ± 6.95	20.48 ± 5.64	< 0.0001
SBJ (cm)	145.61 ± 29.25	160.40 ± 27.19	136.13 ± 26.53	< 0.0001
BAH (sec)	7.86 ± 10.42	14.27 ± 12.31	3.72 ±6.13	< 0.0001
SUP (n/sec.)	16.11 ± 5.60	18.93 ± 3.89	14.30 ± 5.78	< 0.0001
**% Underweight**	92 (35.9%)	44 (44%)	48 (30.7%)	NS
**% Normal**	129 (50.4%)	48 (48%)	81 (51.9%)	
**% Overweight/ obese**	35 (13.7%)	8 (8%)	27 (17.3%)	
**Gender**		-	-	-
Male	100 (39.1%)			
Female	156 (60.9%)			
**Locality**				NS
Town	80 (31.2%)	34 (34%)	46 (29.5%)	
Township	176 (68.8%)	66 (66%)	110 (70.5%)	
**Race**				NS
African	197 (77%)	72 (72%)	125 (80.1%)	
White	59 (23%)	28 (28%)	31 (19.9%)	

NS = Not significant; %BF = percentage body fat; SBJ = standing broad jump; BAH = bent arm hang; SUP = sit-ups.

Table2 shows the mean and standard deviations of physical fitness characteristics for the BMI categories. For all three fitness items, girls in the underweight group performed better than girls in the normal weight group, who performed better than girls in the overweight group. For boys, more or less the same pattern was found, although the differences were (much) smaller. In addition, boys in the normal weight followed by the underweight group were able to jump significantly better than the overweight group. Furthermore, a borderline significant difference was found in bent arm hang with underweight boys being able to hang for more seconds than the boys in the normal and overweight groups. In Table3, the results of the multinomial logistic regression analyses showed a strong and significant positive association between physical fitness and BMI for the underweight girls with high physical fitness scores (OR, 10.69 [95%CI: 2.81-40.73], and for the overweight girls with high physical fitness scores (OR, 0.11 [95%CI: 0.03-0.50]) were found. Non-significant weaker positive relationships between physical fitness and BMI for the underweight boys with the high physical fitness scores (OR, 1.80 [95%CI: 0.63-5.09]), and for the overweight boys with high physical fitness scores (OR, 0.18 [95%CI: 0.02-1.78]) were found. It is clear from the observed associations between physical fitness and BMI that the underweight group performed best.

**Table 2:** Means and standard deviations (sd) of body composition and physical fitness characteristics for the BMI categories

	**Boys**				**Girls**			
	**Underweight**	**Normal Weight**	**Overweight**	**P-value**	**Underweight**	**Normal Weight**	**Overweight**	**P-value**
	**(a)**	**(b)**	**(c)**		**(a)**	**(b)**	**(c)**	
	Mean	Mean	Mean		Mean	Mean	Mean	
	(sd)	(sd)	(sd)		(sd)	(sd)	(sd)	
Standing road (cm)	163.66	164.23	141.88	0.02(ac, bc)**	146.45	139.81	122.93	0.00(ac, bc)**
	(19.72)	(23.14)	(19.56)		(15.98)	(18.38)	(18.74)	
Bent arm hang	16.17	14.03	5.3	0.07	7.17	2.71	0.54	0.00(ab, ac)**
	(11.06)	(13.19)	(10.41)		(8.36)	(4.33)	(1.89)	
Sit ups (n/sec.)	18.82	18.88	19.50	0.9	15.56	14.22	12.37	0.07
	(3.47)	(4.35)	(3.21)		(4.19)	(6.56)	(5.33)	

# n/sec. = number of sit-ups in 30 seconds.

** = significant difference between BMI categories.

**Table 3:** Results of multinomial logistic regression analyses relating BMI categories and physical fitness

		**Girls**				**Boys**			
		Crude OR	95% CI	Adjusted*	95% CI	Crude OR	95% CI	Adjusted*	O95% CI
Underweight	High PF	9.23	2.49-34.21	10.69	2.81-40.73	1.45	0.54-3.88	1.80	0.63-5.19
	Moderate PF	3.87	1.01-14.92	4.08	1.05-15.83	1.36	0.49-3.74	1.33	0.47-3.78
Overweight	High PF	0.16	0.04-0.63	0.11	0.03-0.50	0.21	0.02-1.95	0.18	0.02-1.78
	Moderate PF	0.32	0.11-0.89	0.28	0.09-0.82	0.23	0.02-2.10	0.23	0.02-2.23

* adjusted for race and locality of the schools.

## Discussion

The present study provides data on the prevalence of underweight, normal weight and overweight among adolescents, and the association between body composition and physical fitness separately for boys and girls aged 14 years old adjusted for race and locality in the Tlokwe Local Municipality of the North West Province of South Africa. Body composition is an important component of physical fitness of an individual and provides an indicator of the good well-being [1,17,21,32,46], hence should be emphasized as a way of healthy lifestyle among adolescents in this area [47,48]. The present findings reflect the existence of both underweight (44%; 30.7%) and overweight/obesity (8%; 17.3%) respectively for boys and girls. Additionally, the results showed a strong significant positive association (OR, 10.69[95%CI: 2.81-40.73]) between physical fitness and BMI for underweight girls with high physical fitness scores, and overweight girls with high physical fitness scores (OR, 0.11 [95%CI: 0.03-0.50]).

Consistent with other previous studies in sub-Saharan Africa [3-6], and elsewhere [1,2,46], our findings showed that underweight was higher in boys than in girls on the one hand while on the other hand overweight/obesity was higher in girls than boys. It should be noted that the consequences of both underweight and obesity are reported to be related to decreased physical exercise/work capability and then reduced health-related physical fitness, such as cardiorespiratory fitness, strength and speed of movement [30-34,46]. However, because overweight adolescents with low physical fitness in the study, especially girls tended to perform poorly in physical fitness as such physical activity interventions, or follow-up studies recognizing this relationship particularly in the overweight adolescents are needed [36,48]. Despite that the present sample was less representative of the 2002 South African National Demographics and Health Survey [16], the rapid increasing of underweight and overweight can still be evident.

With regard to the physical fitness, the normal and underweight adolescent boys respectively, significantly outperformed the overweight boys in explosive strength although the normal group performed better than the other groups. Similarly, underweight girls in the present study performed better in explosive strength than the normal and overweight groups. The observed differences may be explained by the fact that both the normal and underweight groups, compared to the overweight group were able to carry an extra load to be moved during weight-bearing tasks [31-34]. Moreover, boys in the present study performed better than the girls and these findings are similar to the previous findings [23,32]. The gender differences in physical fitness performance can be explained in part by gender difference in body composition. It is been reported that boys have greater muscle mass, bone density and less body fat than girls across age groups [17,19,30,34,49]. Previous research studies indicated that excessive fatness have a negative impact on the performance tasks in which the body is projected through space, as in standing broad jump, and on tasks in which the body must be lifted in space, as in bent arm hang [30-32,38].

Additionally, both the underweight boys and girls were able to hang more seconds in bent arm hang than the overweight group. A study by Artero et al. [34] indicated that overweight and obese children can perform equally well or even better than children with normal weight in those muscular fitness tests, and such trend was found in the sit-ups test item especially for boys in the present study. No significant differences were found in sit-ups in the normal, underweight and obese groups in girls. Comparatively, the physical fitness performances of adolescents in this study were poorer than that of the AVENA study [34] and the Republic of Seychelles study [27].

The results showed strong ORs for the relationship between physical fitness and body composition for moderately fit overweight girls. The observed relationship may be explained by many factors, among others high level of physical fitness which may be attributed to household chores and walking distance to and from school [37], but unfortunately such information was not assessed in this study. The results show that children with high physical fitness have lower odds for being overweight than children with low physical fitness, and these findings are consistent with other studies. For example, research findings by Shang et al. [46] revealed that overweight and obese children performed worse in physical fitness exercises compared to normal weight children. Comparable findings from a cross-sectional study in adolescents from the Republic of Seychelles by Bovet et al. [27] reported a strong inverse relationship between physical fitness and excess body weight. In the present study overweight girls with low physical fitness performed worse in physical fitness than the boys and as such these differences may be explained by the fact that the development of BMI for girls is reported to be greater than that of boys from 12 years to 14.9 years [19]. Since the development is greater it might also have more effect on the capability to perform physical fitness which may explain the stronger association found in girls [19,49,50]. From the epidemiological perspectives it is being reported that over-nutrition, which is associated with inadequate physical activity, is assumed to be the product of several risk factors during adolescence [24,26,29], hence the need for strategic intervention.

It should be realized that the current study has some limitations which requires caution in the interpretation of the data. It should be noted that physical fitness is a function of both physical activity and non-modifiable factors such as genetics [19,31], and these factors were however not assessed in the present study and therefore, it was impossible to assess their relative contribution. This study was based on cross-sectional data (baseline measurements) of the PAHLS study, which is set up to be a five year longitudinal study on a group of 14 year-olds who will be followed up until they reach 18 years of age. Available data on measures of strength rather than cardiovascular health is a limitation of the study, which in future studies will be incorporated. In addition, the categorization of adolescents by their BMI may have had an effect on sample size, which as such may have contributed to observed large confidence intervals. Nevertheless, this cross-sectional study will contribute important information about the population of Tlokwe Local Municipality to the body of science. Another limitation of this study might be that the performed analyses are only adjusted for locality of the schools and race. Maturation may one way or the other have affected the results notwithstanding that the study subjects are drawn from a homogeneous age group; unfortunately no reliable data on maturation could be collected within the present study. With the planned longitudinal study we will be able to determine whether the observed prevalence of underweight and overweight as well as its relationship with physical fitness adjusted for other possible confounding factors, such as habitual physical activity, genetics factors, socio-economic status, dietary intake and other modifiable risk factors are consistent over time, and whether the changes in body composition measurements of BMI and %BF are related to changes in physical fitness.

## Conclusion

Our baseline study of 14 year-old high school adolescents residing within the Tlokwe Local Municipality indicates the coexistence of both underweight and overweight. In addition, the results show a strong association between physical fitness and body composition especially in the overweight girls on the one hand, while on the other hand the underweight performed better than the normal weight, and than the overweight. The results show the same trend for boys, but not as strong. The girls were therefore more affected than the boys. As such, from public health perspectives in a country like South Africa with a double paradox of weight status, intervention programs with the objective of low fat mass for overweight and fat-free mass muscular fitness for underweight adolescents should be the start of the beginning, hence well-structured physical fitness program for all.

## Competing interests

All the authors declare that they have no competing interests.

## Authors’ contributions

MAM is the principal investigator of the PAHL study, designed the study, supervised data collection and wrote the paper. RN did the statistical analysis and write-up of the paper. SJM participated in the data collection and provided inputs on the paper. JWRT provided guidance with statistical analysis of the findings and writing of the paper. All the authors participated in the review of the manuscript, read and approved the final manuscript.
